# Characterizing bacterial communities in *Phragmites australis* rhizosphere and non-rhizosphere sediments under pressure of antibiotics in a shallow lake

**DOI:** 10.3389/fmicb.2022.1092854

**Published:** 2022-12-06

**Authors:** Ling Zhang, Junhong Bai, Kegang Zhang, Zhuoqun Wei, Yaqi Wang, Haizhu Liu, Rong Xiao, Milko A. Jorquera

**Affiliations:** ^1^School of Environment, Beijing Normal University, Beijing, China; ^2^School of Chemistry and Chemical Engineering, Qinghai Normal University, Xining, China; ^3^Department of Environmental Engineering and Science, North China Electric Power University, Baoding, China; ^4^College of Environment and Safety Engineering, FuZhou University, Fuzhou, China; ^5^Laboratorio de Ecología Microbiana Aplicada (EMALAB), Departamento de Ciencias Químicas y Recursos Naturales, Universidad de La Frontera, Temuco, Chile

**Keywords:** antibiotics, rhizosphere, bacterial community, sediments, shallow lake

## Abstract

**Introduction:**

Antibiotics are ubiquitous pollutants and widely found in aquatic ecosystems, which of rhizosphere sediment and rhizosphere bacterial communities had certain correlation. However, the response of bacterial communities in *Phragmites australis* rhizosphere and non-rhizosphere sediments to antibiotics stress is still poorly understood.

**Methods:**

To address this knowledge gap, the samples of rhizosphere (R) and non-rhizosphere (NR) sediments of *P*. *australis* were collected to investigate the differences of bacterial communities under the influence of antibiotics and key bacterial species and dominate environmental factors in Baiyangdian (BYD) Lake.

**Results:**

The results showed that the contents of norfloxacin (NOR), ciprofloxacin (CIP) and total antibiotics in rhizosphere sediments were significantly higher than that in non-rhizosphere sediments, meanwhile, bacterial communities in non-rhizosphere sediments had significantly higher diversity (Sobs, Shannon, Simpsoneven and PD) than those in rhizosphere sediments. Furthermore, total antibiotics and CIP were found to be the most important factors in bacterial diversity. The majority of the phyla in rhizosphere sediments were *Firmicutes*, *Proteobacteria* and *Campilobacterota*, while *Proteobacteria*, *Chloroflexi* was the most abundant phyla followed by *Bacteroidota*, *Actinobacteriota* in non-rhizosphere sediments. The dominate factors of shaping the bacterial communities in rhizosphere were total antibiotics, pH, sediment organic matter (SOM), and NH_4_-N, while dissolved organic carbon (DOC), NO_3_-N, pH, and water contents (WC) in non-rhizosphere sediments.

**Discussion:**

It is suggested that antibiotics may have a substantial effect on bacterial communities in *P*. *australis* rhizosphere sediment, which showed potential risk for ARGs selection pressure and dissemination in shallow lake ecosystems.

## Introduction

Antibiotics are widely used in medical treatment, agriculture, breeding, etc., and their superior performance has brought huge benefits. Most antibiotics ingested in humans or animals are not fully absorbed and metabolized, but enter the environment through excrement (Githinji et al., 2011). Some scholars found that higher concentrations of antibiotics were detected in the water and sediments of lakes, due to the discharge of waste water from pharmaceutical factories, sewage treatment plants, hospitals and farm in upstream or surrounding lakes (Xu et al., 2016; Wang et al., 2022).

Pollutants circulate slowly in the lake and are vulnerable to pollutants such as antibiotics due to slower pollutant circulation in the lakes than in other water environments (Nnadozie and Odume, 2019). Aquatic plants, as an important part of the lake ecosystem, also play an important role in the fate of pollutants in lakes (Zhang et al., 2022). Rhizosphere sediment regulates rhizosphere interactions, processes, antibiotics migration and transformation and thus play a vital role in maintaining plant health and ecosystem stability (Li et al., 2022). However, rhizosphere bacterial community also received much attention due to their associations with plant growth and pollution in lake ecosystems (Marschner et al., 2004). The bacterial community in rhizosphere soil can carry out material transformation. In turn, plants transfer their metabolites to the bacterial community in the form of root exudates, thereby affecting the changes of bacterial community (Huang et al., 2020). Pantigoso et al. (2020) presented that aquatic plants were sensitive to external pollution stimulation. However, rhizosphere bacterial communities with maximum microbial activities around plant roots could improve the plant’s response to environmental stress, such as environmental pollution (Otto, 2021). There is multitude of interaction between the numerous microbes and plants in their habitats, which can greatly affect the migration and transformation of pollutants (Thacker and Quideau, 2021). Exogenous pollutants are frequently identified as the most important influencing factors in shaping the bacteria community (Nino Garcia et al., 2016). Therefore, the contaminated environment can result in the difference in microbial community structure and diversity between rhizosphere and non-rhizosphere soils (Thacker and Quideau, 2021; Fortin Faubert et al., 2022).

It has been well documented that plant roots shape and benefit from associated rhizosphere bacterial communities (Edwards et al., 2015; Perez-Jaramillo et al., 2018). What’s more, rhizosphere bacterial communities play a role in supporting pest tolerance (Tronson et al., 2022) and increasing yield (Su et al., 2022). Therefore, the study of rhizosphere bacterial communities is important for the transformation of pollutants, plant health and agricultural production in lake ecosystems. Previous studies have shown that antibiotics can change the structure and function of rhizosphere soil bacterial communities in wetland systems (Tong et al., 2020). Shen et al. (2021) reported that alpha microbial diversity values were decreased for the lettuce non-rhizosphere soil rather than rhizosphere soil with antibiotic exposure in agricultural soil habitat.

However, few studies will involve the response of rhizosphere microorganisms to antibiotics in lake ecosystems, especially, simultaneous responses of bacterial communities to antibiotic stress in rhizosphere and non-rhizosphere sediments are poorly understood. Therefore, our research proposed the following primary objectives were: (1) To identify the differences in bacterial community structure and function under antibiotic exposure in *Phragmites australis* rhizosphere and non-rhizosphere sediments; (2) to explore key bacterial species in *P*. *australis* rhizosphere and non-rhizosphere sediments; and (3) to identify which environmental factors act as the main force for structuring bacterial communities in *P*. *australis* rhizosphere and non-rhizosphere sediments.

## Materials and methods

### Site description

**Figure 1:**
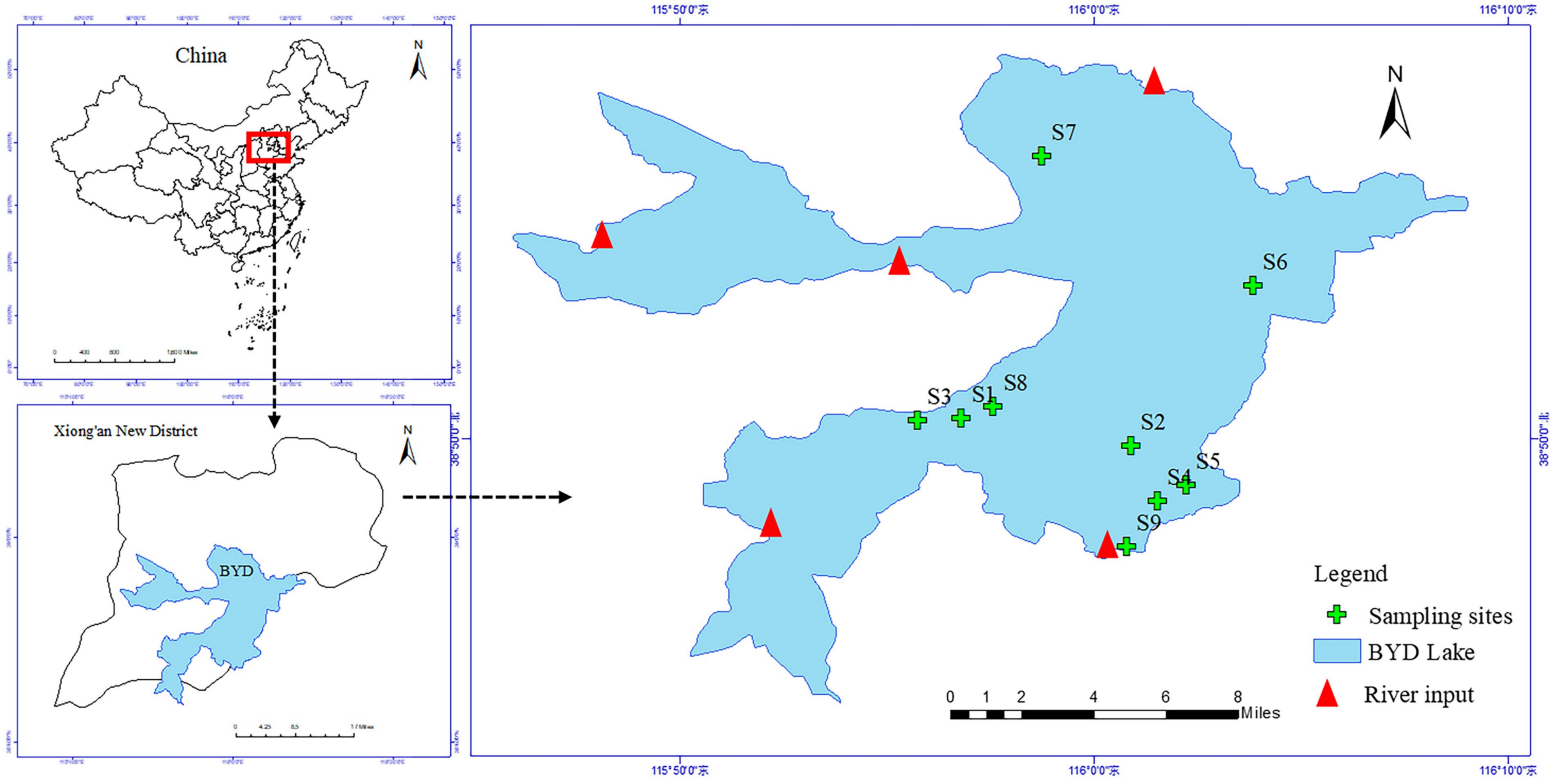
Location map of sampling sites in the Baiyangdian Lake (BYD).

Baiyangdian (BYD) Lake (38°43′ ~ 39°02’N, 115°38′ ~ 116°07′E, Figure 1) is the largest freshwater lake wetland in North China, known as the Kidney of North China, located in the Xiong’an New District of Hebei Province, with a total area of about 366 km^2^. It has a typical temperate semi-arid continental monsoon climate, with the average annual rainfall of 552.7 mm and the evaporation of 1,637 mm, and its water level remains within the range of 1 ⁓ 3 m throughout the year. BYD Lake is covered by various species of aquatic macrophytes, of which *P*. *australis* is one of the dominant emergent macrophytes. The complex aquatic ecological conditions of BYD Lake provides various habitats for aquatic organisms. At present, as an important water body in the Xiong’an New Area, BYD has been given more and more concerns.

### Sample collection and analysis

A total of 9 sampling sites were set up at the vegetation areas covered by *P*. *australis* of BYD Lake. We paired collected 9 samples of rhizosphere sediments (R) and non-rhizosphere soil (NR) surrounding *P*. *australis* in October 2020, respectively (Figure 1). Rhizosphere sediments were obtained from the root zone of *P*. *australis* following the protocol of Huang et al. (2020) by shaking off sediment that was loosely adhering to the roots. For each sampling site, non-rhizosphere sediments without any *P*. *australis* roots were collected by stainless steel static gravity dredger at least 50 cm away from vegetated areas. The collected sediments are stored in three parts to determine antibiotics, physicochemical properties and extract DNA for bioinformatics analysis. All samples were stored in the freezer and send to the laboratory as soon as possible. In the laboratory, one part of samples were freeze-dried in a vacuum freeze dryer, ground in an agate mortar, passed through a 100-mesh sieve, sealed in a plastic bag to physicochemical properties analyses. Another part of samples was stored at −20°C in the dark before antibiotic extraction and extracted and determined as soon as possible. The remaining samples were stored at −80°C to bacterial community analyses.

Zhang et al. (2022) the pH and EC values of the sediments were measured with a pH meter (model ST3100-F, OHAUS Co., Parsippany, NJ, United States, soil to water ratio is 1: 5) and a conductivity meter (HQ40d, Hach Co., Loveland, CO, United States). The water contents (WC) of the sediments were obtained by drying the soils at 105°C for 24 h in an oven. Sediment organic matter (SOM) was measured by dichromate oxidation-colorimetric method (Nelson and Sommers, 1982). The dissolved organic carbon (DOC) and total phosphorus (TP) in the sediments were measured using a Shimadzu TOC meter (TOC-LCPN, Japan) and an inductively coupled plasma atomic emission spectrometer (ICP-MS, Thermo Fisher Scientific, United States), respectively. The NO_3_-N and NH_4_-N in the sediments were measured on an element flow analyzer (AACE, Germany).

### Quality assurance and quality control

The concentrations of nine antibiotics [norfloxacin (NOR), ofloxacin (OFL) and ciprofloxacin (CIP), oxytetracycline (OTC), tetracycline (TC), sulfapyridine (SPD) and sulfadiazine (SDZ), erythromycin (ERM) and roxithromycin (ROM)] was extracted and determinated according to our previous study (Zhang et al., 2022; Supplementary Table S1). Briefly, the antibiotics in the extract were determined by liquid chromatography-mass spectrometry (LC-ESI-MS/MS) in multiple reactive ion detection mode (MRM). An API 4500 QTrap liquid chromatography-mass spectrometer and Waters column (2.1 mm × 100 mm, particle size 1.7 μM) were used for the determination. The best separation conditions were a gradient elution of eluent A (0.1% formic acid ultrapure water) and eluent B (acetonitrile). Tandem mass spectrometry analysis was performed on a Micromass Quattro triple quadrupole mass spectrometer.

The optimized MS/MS parameters for the target antibiotics and antibiotic recoveries in sediment samples were tested, and the detailed results are shown in Supplementary Tables S2, S3. The antibiotic recovery rates ranged from 77.4 to 105.3% in rhizosphere sediments and from 76.9 to 108.6% in non-rhizosphere sediments. The limit of quantification (LOQ) calculated with a signal/noise ratio of 10 was 0.14 ng/g to 1.4 ng/g for rhizosphere sediments, and 0.4 ng/g to 2.6 ng/g for non-rhizosphere sediments.

### DNA extraction and illumina sequencing

The FastDNA® SPIN kit for soil (MP Biomedicals, Solon, OH, United States) was used to extract DNA from 0.5 g homogenized soil. The concentration and purity of the DNA were determined with NanoDrop 2000 UV–vis spectrophotometer (Thermo Fisher Scientific, Wilmington, United States). Polymerase chain reaction (PCR) amplification was performed using the universal primers 338F (ACTCCTACGGGAGGCAGCAG) and 806R (GGACTACHVGGGTWTCTAAT) designed against the V3-V4 region of bacterial 16S rRNA gene in an ABI GeneAmp® 9,700 PCR thermocycler (ABI, CA, United States). Amplicon sequencing was performed using the Illumina MiSeq platform at the Shanghai Majorbio Bio-pharm Technology Co., Ltd., in China.

The PCR amplification of 16S rRNA gene was performed as follows: initial denaturation at 95°C for 3 min, followed by 30 cycles of denaturing at 95°C for 30 s, annealing at 55°C for 30 s and extension at 72°C for 45 s, and single extension at 72°C for 10 min. The PCR was carried out in triplicates in a 20 μL reaction mixture containing 5× FastPfu Buffer, 2.5 mM dNTPs, 5 μM of each forward and reverse primer, FastPfu DNA Polymerase, BSA and 10 ng template DNA. PCR products were purified from a 2% agarose gel using an AxyPrep DNA Gel Extraction Kit (Axygen, United States), quantified using a QuantiFluor™-ST Blue Fluorescence Quantitative System (Promega Company), and pooled together for library preparation using a TruSeqTM DNA Sample Prep Kit (Illumina, San Diego, CA, United States) following the manufacturer’s recommendations. Sequencing was performed using Illumina’s Miseq PE300 platform. After splitting the PE reads obtained by MiSeq sequencing, the double-end reads were first quality-controlled and filtered according to the sequencing quality, and spliced according to the overlap relationship between the double-end reads to obtain optimized data after quality control splicing. Then, the sequence noise reduction method (DADA2/Deblur, etc.) is used to process the optimized data to obtain amplicon sequence variants (ASVs) representative sequence and abundance information. Based on ASV representative sequence and abundance information, bioinformatics analysis can be performed. Alpha diversities were used in our study to evaluate bacterial diversities (i.e., Shannon), abundance (i.e., Sobs), bacterial evenness (i.e., Simpsoneven), and phylogenetic diversities (PD). The sequence data were submitted to NCBI Sequence Read Archive with the accession number PRJNA899316.

### Statistical analysis

SPSS software was used for descriptive statistical analysis of antibiotic contents in different environmental media. One-way ANOVA analysis was performed to test the differences in the concentrations of nine antibiotics among different sediments. The Random Forests model was used to identify the importance of environmental factors and the significance of the models and cross-validated *R*^2^ values were assessed with 1,000 permutations of the response variable using the A3 package R. The significance level was described as *p* < 0.05, *p* < 0.01, or *p* < 0.001. These statistical analysis were performed using SPSS 24.0 for Windows and R (v4.1.1).^1^

Alpha diversity was calculated at the ASV levels. ANOVA with Duncan’s test was performed to determine the differences in bacterial alpha diversities. Beta diversity expressed as principal coordinate analysis (PCoA) based on the Bray-Curtis distance matrix. The Spearman’s correlation will be considered to be significant if the value of *p* is <0.05. Linear discriminant analysis effect size (LEfSe) analysis was conducted to identify the significantly changed bacterial and the relationships between sediments bacterial communities and the environmental variables were determined using canonical correlation analysis (CCA). These analyses were conducted using the vegan package R in free online platform of Majorbio Cloud Platform^2^ (Yu et al., 2022).

## Results

### Antibiotics characteristics of *Phragmites australis* rhizosphere and non-rhizosphere sediments

**Figure 2:**
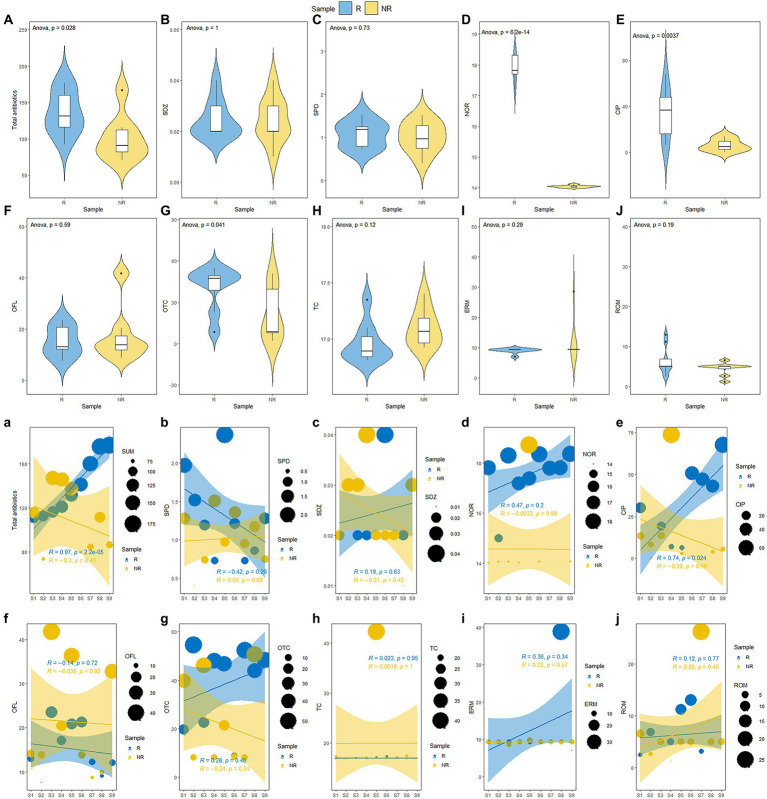
**(A–J)** Changes of antibiotic concentration *P*. *australis* rhizosphere and non-rhizosphere sediments and **(a-j)** along a gradient of increasing antibiotic concentration.

The changes in antibiotic and physicochemical properties of *P*. *australis* rhizosphere and non-rhizosphere sediments properties are shown in Figure 2 and Supplementary Table S4. The total antibiotic concentrations in rhizosphere sediments ranged from 92.62 ng/g to 275.57 ng/g, with the average concentration of 153.45 ng/L, while that varied from 70.4845 ng/g to 285.72 ng/g, with the average concentration of 126.80 ng/g in non-rhizosphere sediments. Overall, rhizosphere sediments significantly enriched the antibiotics (*p* < 0.05, Figure 2A). Similarly, the concentrations of NOR (17.55 ± 0.98 ng/g) and CIP (53.97 ± 30.59 ng/g) were significantly higher in rhizosphere sediments than that in non-rhizosphere sediments (NOR: 14.62 ± 1.55 ng/g; CIP: 6.18 ± 4.84 ng/g) (*p* < 0.05, Figure 1D), while other antibiotics such as SPD, SDZ, OTC, TC, ERM, and ROM showed no significant difference between rhizosphere and non-rhizosphere sediments (*p* > 0.05, Figures 1B,C,G–J). However, significantly higher NH_4_-N, NO_3_-N, WC, and TP were observed in rhizosphere sediments than that in non-rhizosphere sediments (*p* < 0.05, Supplementary Table S4), while DOC and SOM were significantly higher in non-rhizosphere sediments (*p* < 0.05, Supplementary Table S4). The pH varied from 7.35 to 7.84 in non-rhizosphere sediments and from 7.45 to 7.54 in rhizosphere sediments (*p* > 0.05, Supplementary Table S4).

Our results showed that the total antibiotics were accumulated in *P*. *australis* rhizosphere sediments. Accordingly, we ranked the concentrations of total antibiotics in rhizosphere sediments from low to high (from S1 to S9, Figure 1A). The results showed that the total antibiotics showed various trends in the rhizosphere and non-rhizosphere sediments. The total antibiotics in *P*. *australis* rhizosphere sediments showed a significant linear increase from S1 to S9 (*R* = 0.97, *p* < 0.001, Figure 2A) and no significant (*p* > 0.05, Figure 2A) relationships in *P*. *australis* non-rhizosphere sediments with inflection point at S3 (147.12 ng/g). Similarly, there was a significant linear increase in the CIP concentration in rhizosphere sediments from S1 to S9 (*R* = 0.74, *p* < 0.05, Figure 2E) Besides, the concentrations of NH_4_-N, NO_3_-N, DOC, WC, and TP showed higher levels in non-rhizosphere sediments than those in rhizosphere sediments from S1 to S9, except for SOM. What’s more, TP concentration showed a linear increase from S1 to S9 in non-rhizosphere sediments (*R* = 0.65, *p* = 0.06, Supplementary Figure S1). A different changing trend with a decrease before increasing was observed for NH_4_-N (*R* = −0.39, Supplementary Figure S1) and WC in non-rhizosphere sediments (*R* = −0.20, Supplementary Figure S1).

### Effects of antibiotics on bacterial diversity

**Figure 3:**
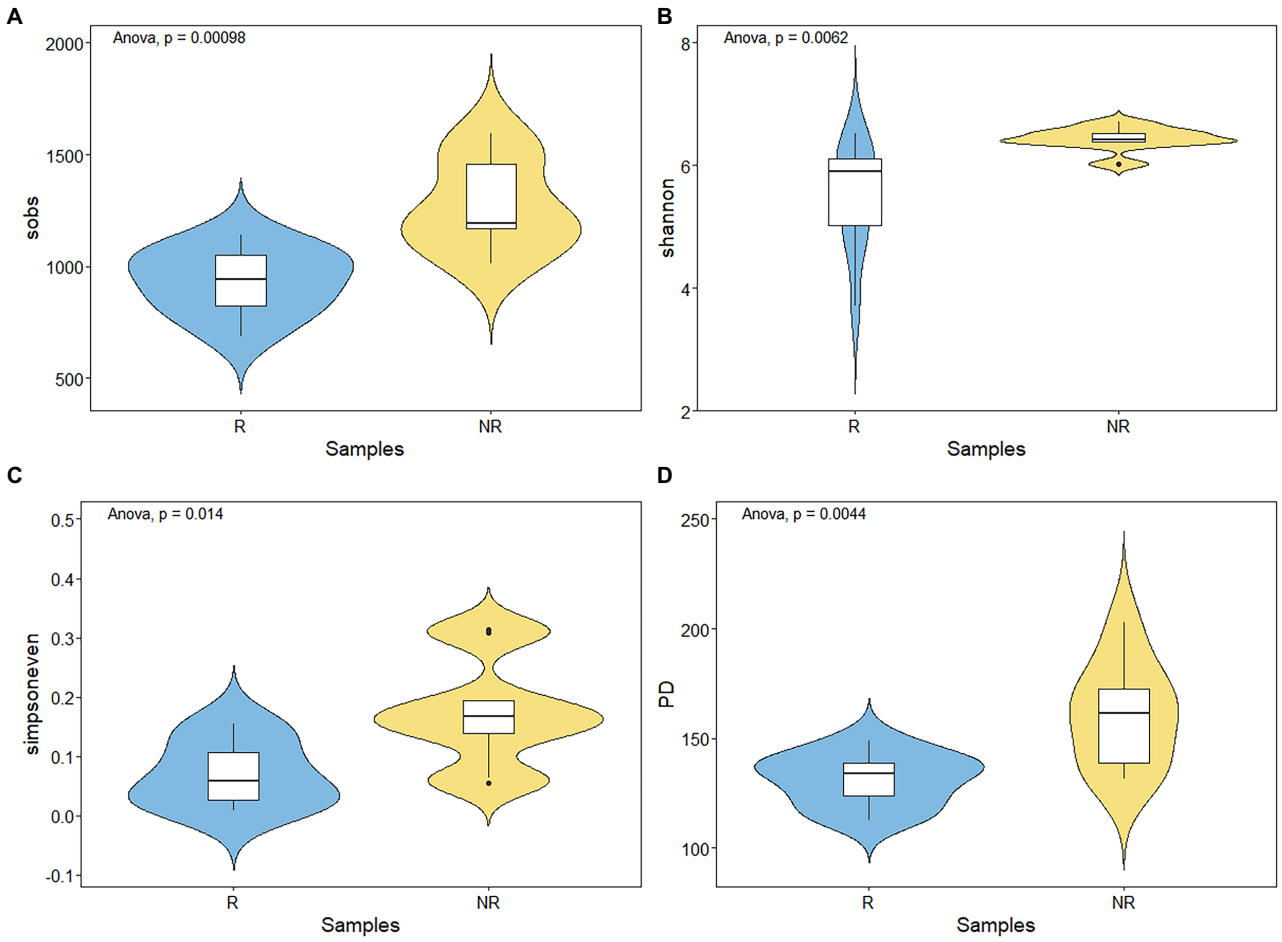
**(A–D)** Variations of bacterial alpha diversity in rhizosphere and non-rhizosphere sediments.

**Figure 4:**
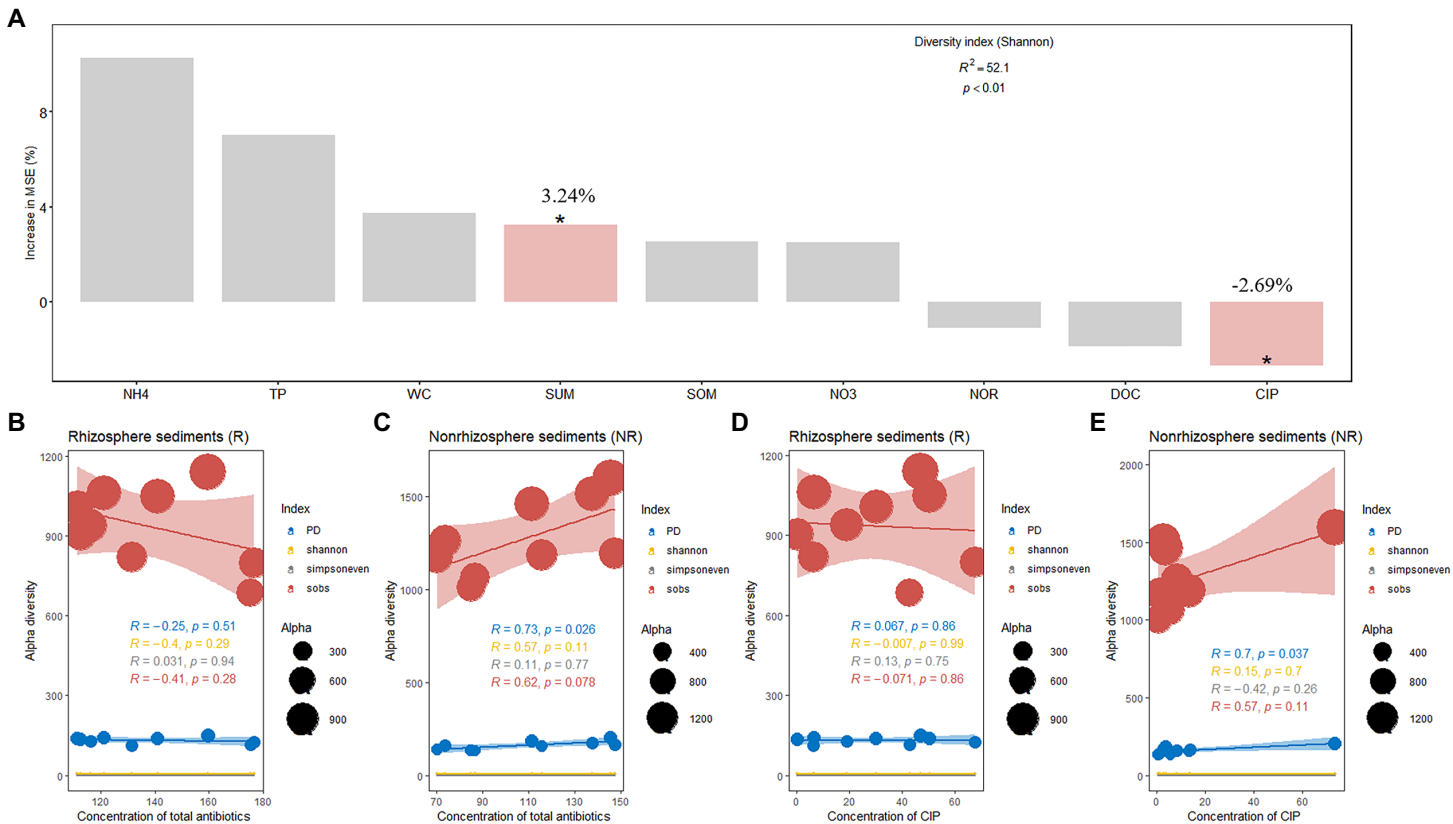
**(A)** Random forest indicating the potential factors of variation in bacterial alpha diversity and **(B–E)**, Effect of antibiotic concentration on bacterial alpha diversity.

A total of 468,192 and 543,524 bacterial sequences were detected, 1,232 and 934 ASVs were clustered in rhizosphere and non-rhizosphere sediments, respectively. As shown in Figure 3. We evaluated the community richness (Sobs indices), community diversity (Shannon indices), community evenness (Simpsoneven indices) and phylogenetic diversity (PD) to further compare the bacterial community structure in rhizosphere and non-rhizosphere sediments. The alpha diversity of the bacterial communities in different sediment samples also exhibited certain differences (Figure 3). Results showed that non-rhizosphere sediments had significantly higher diversity (Sobs: 1271.22 ± 192.56, Shannon: 6.44 ± 0.18, Simpsoneven: 0.17 ± 0.08 and PD: 160.57 ± 22.37) compared with rhizosphere sediment (Sobs: 943.33 ± 137.77, Shannon: 5.48 ± 0.84, Simpsoneven: 0.071 ± 0.052 and PD: 131.16 ± 11.33) (*p* < 0.05, Figure 3). Correlation analysis results showed that bacterial Sobs indices (*r* = −0.67, *p* < 0.05, Supplementary Figure S2) and PD indices (*r* = −0.80, *p* < 0.01, Supplementary Figure S2) were significantly negatively correlated with NO_3_-N concentrations in rhizosphere sediments, while positively correlated with NH_4_-N (*r* = 0.72, *p* < 0.05) and CIP (*r* = 0.65, *p* < 0.05) in non-rhizosphere sediments (Supplementary Figure S2). In contrast, Shannon indices were positively correlated with OFL (*r* = 0.72, *p* < 0.05, Supplementary Figure S2), and simpsoneven indices were negatively correlated with SOM (*r* = −0.75, *p* < 0.05, Supplementary Figure S2) and positively correlated with NH_4_-N, ROM and OFL (*r* > 0.71, *p* < 0.05, Supplementary Figure S2).To disentangle the potential main factor for bacterial diversity under antibiotics stress, we explored the relative importance of environmental factors to influence the bacterial diversity by the random forest analysis (Figure 4A). Total antibiotics (SUM) and CIP were found to be the most important factors in bacterial diversity. Meanwhile, the bacterial diversity (PD) exhibited strong linear correlation with total antibiotics (*R* = 0.73, *p* < 0.05, Figure 4C) and CIP (*R* = 0.70, *p* < 0.05, Figure 4C) in non-rhizosphere sediment. However, no significant differences (*p* > 0.05) in Sobs, Shannon and Simpsoneven indices were observed both in rhizosphere and non-rhizosphere sediments as the antibiotic concentration increased (Figures 4B,C).

The variations in bacterial communities between rhizosphere and non-rhizosphere sediments were assessed by principal coordinate analysis (PCoA) based on Bray-Curtis distance measure (Supplementary Figure S3). Rhizosphere sediments showed significant differences in bacterial beta diversity compared with non-rhizosphere sediments (*p* < 0.05, Supplementary Figure S3), and The PC1 and PC2 axes explained 31.16% of the variation in the bacterial community. Along the PC2 axis, the bacterial community in rhizosphere sediments was separated from that in non-rhizosphere sediments.

### Bacterial community changes in rhizosphere and non-rhizosphere sediments

**Figure 5:**
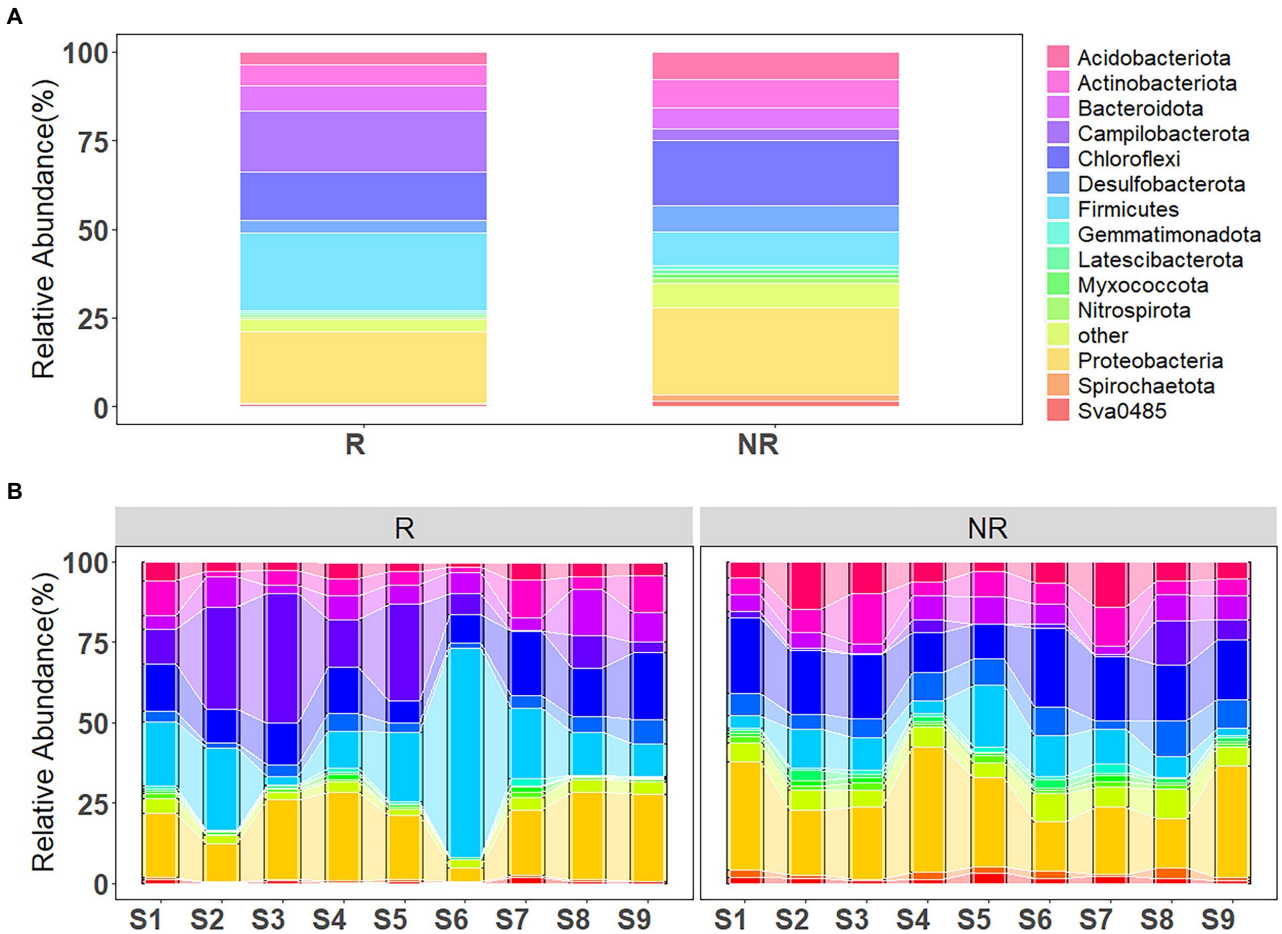
**(A)** Bacterial community composition in rhizosphere and non-rhizosphere sediments and **(B)** along the concentration gradient of antibiotics.

As shown in Figure 5A, the ASVs were assigned to 53 phyla and 1,593 species for rhizosphere bacterial communities, and 62 phyla and 1714 species for non-rhizosphere bacterial communities. The majority of the bacterial ASVs were classified as *Firmicutes* (21.80% of the total relative abundance), *Proteobacteria* (19.94%), *Campilobacterota* (17.20%), *Chloroflexi* (13.56%), *Bacteroidota* (7.19%), *Actinobacteriota* (5.85%) at the phylum level in rhizosphere sediments, while *Proteobacteria* (24.75%), *Chloroflexi* (18.60%), *Firmicutes* (9.34%), *Acidobacteriota* (7.91%), *Actinobacteriota* (7.88%), *Desulfobacterota* (7.36%) dominated the sample bacterial community in non-rhizosphere sediments.

Variations in bacterial community composition with increasing antibiotic concentrations (from S1 to S9) is shown in Figure 5B. *Proteobacteria*, *Chloroflexi* and *Actinobacteriota* were the main phyla in sediment samples, with an increasing tendency, while *Firmicutes* and *Campilobacterota* demonstrated a slight decreasing tendency, in the rhizosphere sediments from S1 to S9 sampling sites. However, different trend has been observed from bacterial community composition in non-rhizosphere sediment samples. *Proteobacteria*, *Chloroflexi* and *Actinobacteriota* showed a decreasing tendency. Whereas, *Firmicutes* and *Campilobacterota* posed an increasing tendency from S1 to S9 sampling sites.

**Figure 6:**
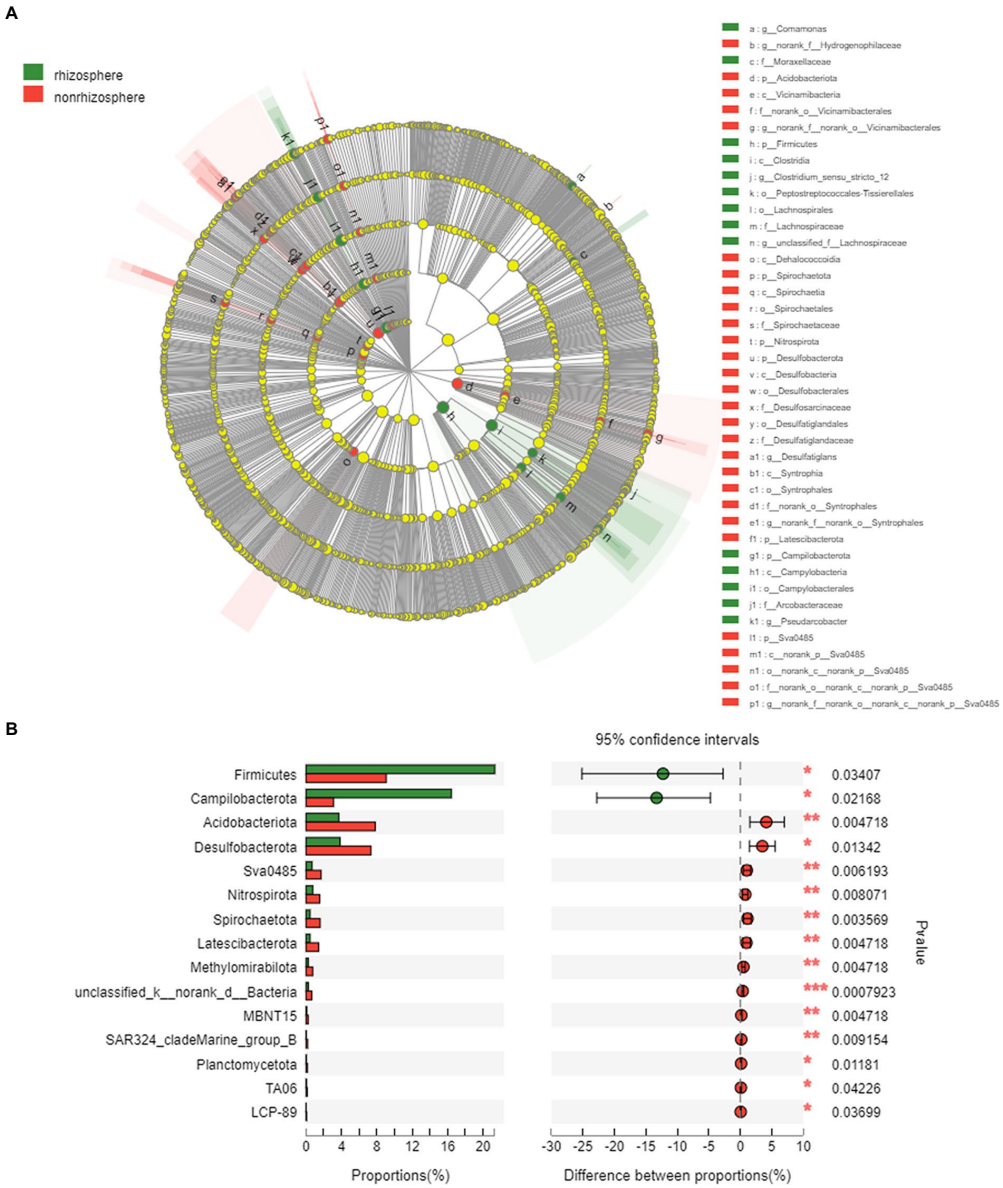
**(A)** LEfSe analysis of bacteria on rhizosphere and non-rhizosphere sediments (LDA>3.0) and **(B)** Key differential species in rhizosphere and non-rhizosphere sediments.

The LEfSe was performed to identify high-dimensional biomarker taxa with significantly different bacterial abundances in rhizosphere and non-rhizosphere sediments (Figure 6). In total, 14 and 28 bacterial abundant taxa were identified with an LDA threshold of 3.5 in rhizosphere and non-rhizosphere sediments, respectively (Supplementary Figure S5). The LEfSe results revealed that the specific bacterial biomarkers in rhizosphere sediments were phylum *Firmicutes* (including the class *Clostridia* and genera *unclassified_f__Lachnospiraceae*), and *Campilobacterota* (from phylum to genus *Pseudarcobacter* and class *Campylobacteria*). For the non-rhizosphere sediments, specific bacterial biomarkers were *Acidobacteriota* (from phylum to its genus *norank_f__norank_o__Vicinamibacterales*), *Desulfobacterota* (from phylum to its genus *norank_f__norank_o__Syntrophales and Desulfatiglans*), and *Vicinamibacteria* class, *Desulfobacteria* class.

### Relationships between the soil environment and the bacterial communities

**Figure 7:**
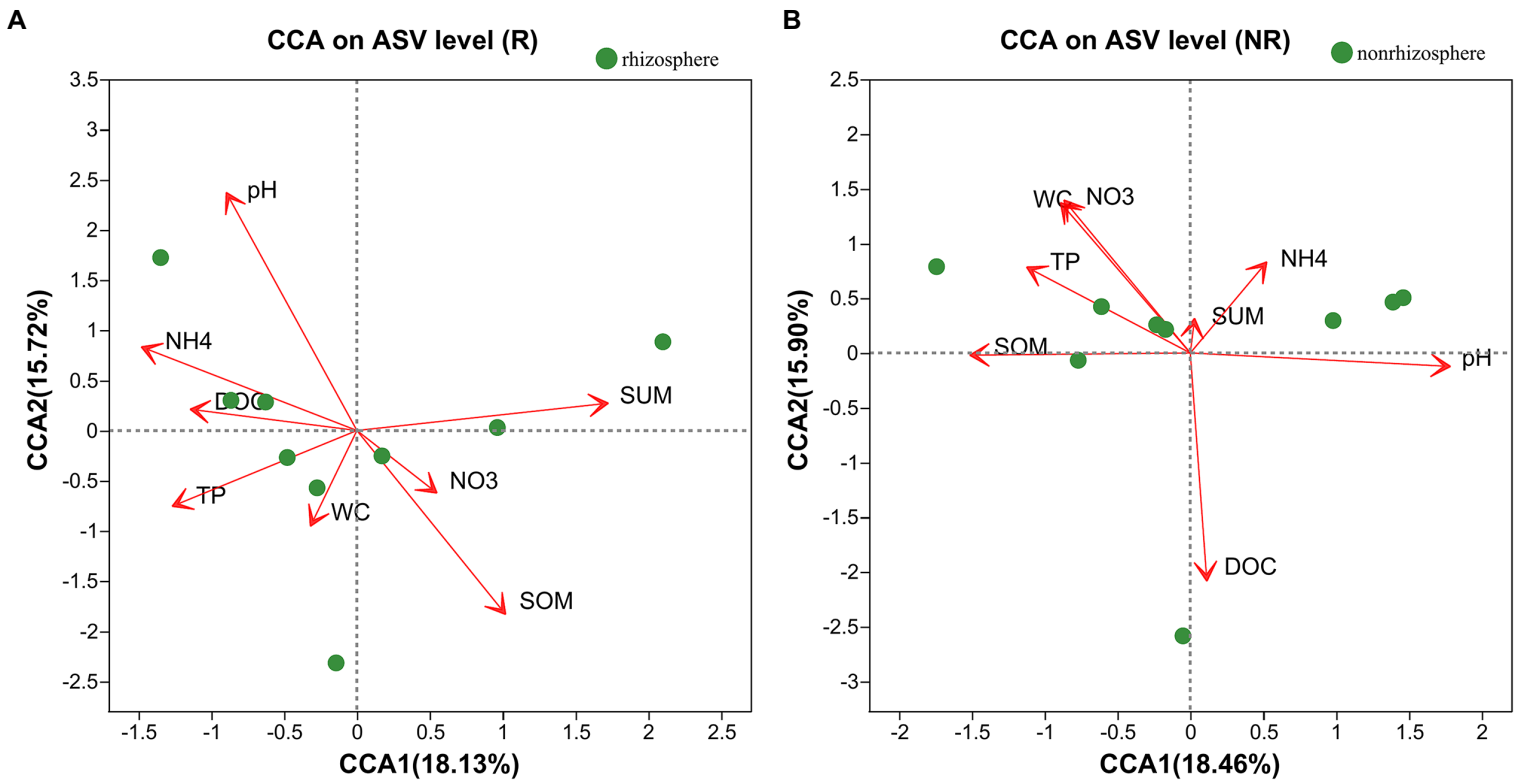
**(A,B)** CCA analysis between bacterial communities and environmental factors.

The relationships between the bacterial community and rhizosphere or non-rhizosphere sediments environmental factors were analyzed by CCA (Figures 7A,B). As for rhizosphere sediments (Figure 7A), the first CCA axis was mainly positively correlated with total antibiotics and negatively correlated with pH, NH_4_-N and DOC, while the second CCA axis was mainly positively correlated with pH and total antibiotics and negatively correlated with TP and SOM. However, the first CCA axis was mainly positively correlated with NH_4_-N, pH and DOC and negatively correlated with SOM, TP, NO3 and WC in non-rhizosphere sediments (Figure 7B).

**Figure 8:**
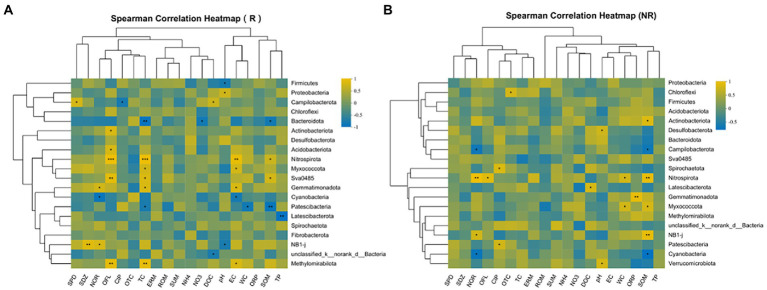
**(A,B)** Correlation between environmental factors and microorganisms at phylum level.

Compared with non-rhizosphere bacterial communities, the rhizosphere bacterial communities had a more regular correlation with environmental factors in the correlation heatmap analysis (Figure 8). For rhizosphere bacterial communities, the relative abundance of the two most dominant phyla *Firmicutes* (*r* = −0.68) and *Proteobacteria* (*r* = 0.68) significantly correlated with sediment pH (*p* < 0.05). Nevertheless, the relative abundance of *Campilobacterota* had significant correlations with DOC (*r* = 0.67, *p* < 0.05), CIP (*r* = −0.7, *p* < 0.05) and SPD (*r* = 0.76, *p* < 0.05). Specifically, the relative abundance of *Bacteroidota* had significant negative correlations with TC (*r* = −0.82, *p* < 0.01), NO_3_-N (*r* = −0.72, *p* < 0.05) and SOM (*r* = −0.71, *p* < 0.05). However, OFL and TC showed significant correlations with *Nitrospirota* (*r* > 0.91, *p* < 0.001), *Sva0485* (*r* > 0.76, *p* < 0.05), *Methylomirabilota* (*r* > 0.85, *p* < 0.01), *Latescibacterota* and TP was significantly correlated with *r* = −0.86 in rhizosphere sediment, specifically. *Patescibacteria* (*r* = −0.85, *p* < 0.01), *Sva0485* (*r* = 0.71, *p* < 0.05) was significantly correlated to SOM.

In non-rhizosphere, NOR and SOM were significantly negatively correlated with the relative abundances of *Cyanobacteria* (*r* > −0.72, *p* < 0.05) and *Campilobacterota* (*r* > −0.68, *p* < 0.05), while significantly positively correlated with the relative abundances of *Nitrospirota* (*r* > 0.83, *p* < 0.01). Moreover, *Chloroflexi* was significantly positively correlated with OTC (*r* = 0.7, *p* < 0.05). Besides, the relative abundances of *Nitrospirota* were significantly positively correlated with OFL (*r* = 0.78, *p* < 0.05) and WC (*r* = 0.68, *p* < 0.05). Similarly, the relative abundances of *Actinobacteria* and *Desulfobacterota* were significantly positively correlated with SOM (*r* = 0.76, *p* < 0.05) and pH (*r* = 0.67 *p* < 0.05), respectively.

## Discussion

### Changes in bacterial communities under antibiotics stress

The rhizosphere is the critical zone where roots access water and nutrients, and interact intimately with the physical, chemical, and biological components of the soil or sediments (Wang et al., 2020). Rhizosphere processes have an important role in the fate of nutrient pollutants in the rhizosphere environment (Lynch, 2019). Rhizosphere bacterial communities also are important for plant growth by influencing SOM breakdown and improving pollutant accumulation by plant roots in the rhizosphere (Marschner et al., 2004; Reinhold-Hurek et al., 2015). Our results showed that the CIP and NOR and total antibiotics in rhizosphere sediments were higher than that in non-rhizosphere sediments, which is consistent with previous result by Chen et al. (2018). This indicated that the accumulation of antibiotics by plant roots in the rhizosphere was stronger due to rhizosphere bacteria may stimulate some transporter protein (Souza et al., 1999). However, CIP and NOR are more easily deposited in sediments due to the adsorption of suspended matter in water and organic matter in sediments (Gong et al., 2012; Zhang et al., 2022). Therefore, the accumulation of CIP and NOR in aquatic plants mainly occurs in roots (Hoang et al., 2013). However, the degradation of microorganisms in rhizosphere was not strong enough to cause a significant difference between rhizosphere and non-rhizosphere sediments (Song et al., 2020).

Our results showed that the richness, diversity, evenness and phylogenetic diversity of bacterial community in rhizosphere and non-rhizosphere sediments were significantly affected by antibiotics (such as NOR, OFL TC, OTC, etc., Figure 8). Previous studies have also confirmed that the alterations in composition and diversity of bacteria were caused by pollutants from the effluent discharge (Pascual-Benito et al., 2020). Moreover, alpha diversity of bacterial communities observed in non-rhizosphere sediments was significantly higher than that in rhizosphere sediments (Figure 3), which indicates that different root zones have certain effects on microbial diversity. Zhou et al. (2022) also reported that habitat (rhizosphere or bulk) accounted for the greatest amount of variation in bacterial community composition, overriding the importance of environmental interference (such as seasonal and flooding conditions) on these bacterial communities. In our study, the main factors causing the differences of microbial diversity in rhizosphere and non-rhizosphere sediments may be the antibiotics mentioned above and the physicochemical properties of sediments, which could change bacterial community diversity (Zhang et al., 2022).

Rhizosphere and non-rhizosphere sediments have very distinct bacterial communities and specific bacterial biomarkers (Figure 6), which are the integrated result of many different selection factors (Carelli et al., 2000). These factors include the physical and chemical properties of the sediments (e.g., DOC, SOM and pH, etc., Figure 7) and environmental factors such as pollutants. In rhizosphere sediments, the concentrations of total antibiotics, NH_4_-N, NO_3_-N, WC and TP were significantly higher than those in non-rhizosphere sediments, while DOC and SOM were significantly higher in non-rhizosphere sediments (Supplementary Table S4). Previous studies reported that rhizosphere microorganisms are affected by the sediment properties, such as pH (Han et al., 2020). Therefore, the significant change in sediment properties could cause the variations in the bacterial community composition in current study. Marschner et al. (2004) also presented similar results that rhizosphere soil bacterial communities can be influenced by soil chemical and physical properties. As a result, *Firmicutes*, *Proteobacteria* were the most abundant phyla followed by *Bacteroidota*, *Actinobacteriota* in rhizosphere sediments, while *Proteobacteria*, *Chloroflexi*, *Firmicutes*, *Acidobacteriota* were dominated bacterial phyla in non-rhizosphere sediments (Figure 5).

### Links between sediment bacterial communities and environmental factors

The rhizosphere sidiment is important zone where sediments interact with bacterial communities, and environmental factors are responsible for the shaping of bacterial communities (Wang et al., 2020). It has been previously reported that root exudates from plant rhizosphere sedimentscan alter the chemical composition of rhizosphere sediments and affect the behavior of contaminants (Chen et al., 2018). Our result showed that major environmental factor affecting bacterial diversity in rhizosphere were SOM, pH, and total antibiotics (Figure 7A) and in non-rhizosphere sediment were DOC, pH, WC and NO_3_-N (Figure 7B). What’ more, total antibiotics was accumulated in rhizosphere sediment. This is consistent with Katoh et al. (2016) study that the rhizosphere sediments increase the availability of pollution by reducing the rhizosphere pH.

The nutrients provided by root exudates can enrich the bacterial community in rhizosphere sediments and may promote the biodegradation of pollutants (2016). However, in our study this phenomenon is not obvious, as we mentioned earlier, may be the effect of antibiotic bioaccumulation greater than biodegradation. It has been reported that environmental factors were the main driver of bacterial communities (Song et al., 2020). NOR, OFL, and CIP showed strong significant correlations with the dominated bacterial phyla (*p* < 0.05; Figure 8). This could be explained by the fact that these antibiotics (i.e., NOR, OFL, and CIP) are more likely to be retained in sediments (Gong et al., 2012) and exert selective pressure on microorganisms (Gong et al., 2012; Karkman et al., 2019). Additionally, We also found that WC and SOM were significantly related to the dominated bacterial phyla (*p* < 0.05, Figures 7, 8), which is also identified by previous result that moisture and organic matter as significant factors controlled the variation in bacterial communities in the aquatic environment (Chen et al., 2019; Song et al., 2020). Although Song et al. (2020) reported that pH and EC played the most significant role in shaping bacterial communities. However, in current study, pH and EC presented no significant correlation with all phyla in non-rhizosphere sediments and significant correlation with several phyla in rhizosphere sediments, which might explain the positive association of oxygen regulation by bacterial communities (Huang et al., 2020) and root exudates (Lv et al., 2020).

## Conclusion

This study investigated the differences in bacterial community and key bacterial species as well as their main influencing factors in rhizosphere and non-rhizosphere sediments. Our study highlighted the antibiotics remarkably altered the diversity and composition of bacterial communities between *P*. *australis* rhizosphere and non-rhizosphere sediments. More antibiotics were accumulated in *P*. *australis* rhizosphere sediments, while non-rhizosphere sediments had higher bacterial diversity, which may be mainly influenced by total antibiotics and ciprofloxacin (CIP). The main environmental factors affecting bacterial community structure in rhizosphere and non-rhizosphere sediments are different. Total antibiotics, pH and SOM played essential roles in shaping the bacterial communities in *P*. *australis* rhizosphere sediments, while DOC, NH_4_-N, pH and WC could be responsible for the variations bacterial communities in non-rhizosphere sediments. Meanwhile, rhizosphere sediments showed higher potential risks for ARGs selection pressure and dissemination. Overall, these findings suggest that antibiotics significantly changed diversity and structure of bacterial communities, which indicates the utmost importance of developing corresponding control, monitoring, management strategies for antibiotics pollution. Further studies should be carried out to explore selective pressure of antibiotics on bacterial communities from sediments to waters.

## Data availability statement

The original contributions presented in the study are included in the article/Supplementary material, further inquiries can be directed to the corresponding author.

## Author contributions

LZ: writing—investigation and original draft. JB: funding acquisition, and writing—review and editing. KZ, RX, and MJ: review and editing. ZW, YW, and HL: writing—review and editing. All authors contributed to the article and approved the submitted version.

## Funding

This study was financially supported by Projects of International Cooperation and Exchanges NSFC-ANID Fund (number 51961125201 in China and code NSFC190012 in Chile). Partial support was also provided by the Fundamental Research Funds for the Central Universities and the Interdisciplinary Research Funds of Beijing Normal University.

## Conflict of interest

The authors declare that the research was conducted in the absence of any commercial or financial relationships that could be construed as a potential conflict of interest.

## Publisher’s note

All claims expressed in this article are solely those of the authors and do not necessarily represent those of their affiliated organizations, or those of the publisher, the editors and the reviewers. Any product that may be evaluated in this article, or claim that may be made by its manufacturer, is not guaranteed or endorsed by the publisher.

## Footnotes

^1^ https://www.r-project.org/

^2^ https://cloud.majorbio.com/
